# Cryogenic Blackbody Calibrations at the National Institute of Standards and Technology Low Background Infrared Calibration Facility

**DOI:** 10.6028/jres.099.008

**Published:** 1994

**Authors:** R. U. Datla, M. C. Croarkin, A. C. Parr

**Affiliations:** National Institute of Standards and Technology, Gaithersburg, MD 20899-0001

**Keywords:** blackbody calibrations, cryogenic blackbodies, cryogenic radiometer, electrical substitution radiometry, low background infrared radiation calibrations, radiomclry

## Abstract

The Low Background Infrared Calibration Facility (LBIR) at the National Institute of Standards and Technology has been in operation for calibration measurements of the radiant power emitted from infrared radiation (IR) sources, such as cryogenic blackbodies, for more than 2 years. The IR sources are sent to NIST by customers from industry, government, and university laboratories. An absolute cryogenic radiometer is used as the standard detector to measure the total radiant power at its aperture. The low background is provided by a closed cycle helium refrigeration system that maintains the inner parts of the calibration chamber at 20 K. The radiance temperature of the blackbody is deduced from the measured power and compared with the blackbody temperature sensor data. The calibration procedures and data analysis are illustrated using the measurements of a typical blackbody.

## 1. Introduction

**Fig. 1:**
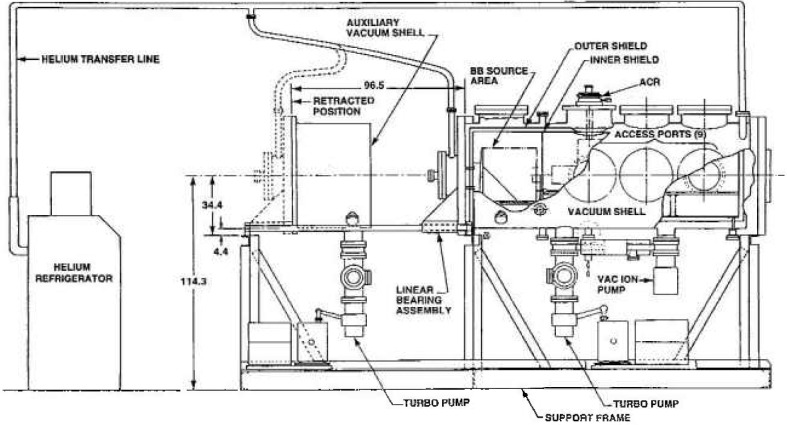
LBIR chamber with partial cutaway showing the major features of the apparatus.

The national need to establish a primary standard reference for calibrating infrared sensors led to the establishment, in 1989, of the Low Background Infrared Calibration Facility (LBIR) at the National Institute of Standards and Technology. This facility has been serving a variety of users from industry and government laboratories by providing calibrations of cryogenic blackbody sources. These sources are used by the customer for calibrations of their infrared sensor systems. An absolute cryogenic radiometer (ACR) of the electrical substitution type has been developed as the standard reference detector for the LBIR calibrations. The ACR measures the total radiant power incident on its precision aperture. Its operating range for radiant power measurements is 20 nW to 100 μW with an expanded uncertainly of ± 1% (95% confidence level). The physical description of the ACR and its characterization as an absolute detector for radiant power measurements have been described in Ref. [1], The LBIR chamber is shown in Fig. 1.

The cylindrical vacuum tank housing the ACR detector and the customer blackbody source is made of stainless steel and is 152 cm long and 60 cm in diameter. Two shields made of copper (shown in the cutaway portion of Fig. 1) separated by a 2.54 cm gap are cooled by a helium refrigerator which circulates cooled helium gas (15 K) through copper lines vacuum brazed to these shields. The inner shield operates at 20 K and provides the low thermal radiation background inside the chamber. The inner surface of the inner shield has a highly absorbent black coating of IITRI MH-2200 paint^1^ to reduce scattered radiation. The ACR can be placed in any of the three ports of the LBIR chamber, which are located at distances of approximately 30 cm, 65 cm, and 100 cm from the blackbody source under test. The placement of the ACR can be chosen so that the radiant power at its aperture is in the ACR measurement range. A complete description of the facility can be found in a previous publication [3].

The general requirements for blackbody calibrations are described in Sec. 2 of this article. The procedures for handling the cryogenics of the calibration setup and the procedures adopted for measurements and analysis are described in Sec. 3. A recent blackbody calibration illustrates the procedures. The blackbody in question has two sensors and is referred to as BB in this paper.

## 2. Calibration Requirements

Calibration requirements fall into two categories: 1) needs established by the customer and 2) needs of the MST LBIR facility. A typical customer requirement for the BB is as follows: 1) measure the radiant power at the ACR aperture for the blackbody temperature range of 200 K to 400 K in increments of 25 K for the source aperture diameter of 650 μm and 2) calibrate the temperature sensors of the source by comparing with the deduced radiance temperature. NIST issues guidelines to the customer for preparing the black-body source to conform to the vacuum and allowable contaminant level specifications of the LBIR chamber. The NIST requirement specifies that the partial pressure of hydrocarbons (beyond approximately 45 u) should not exceed 1.33 × 10^−8^ Pa for a total base pressure of 1.33 ×10^−6^ Pa. Other requirements are described in detail in Ref. [3], NIST also supplies the customer with a mounting plate and guidelines for installing the plate on the blackbody source [3].

## 3. Calibration Procedures

### 3.1 Cryogenic Apparatus and Preparation for Measurements

The blackbody is integrated into the LBIR chamber after testing its vacuum integrity. Cryo-conditioning of the LBIR chamber follows with the inner shield of the chamber being cooled to approximately 20 K and the outer shield being cooled to approximately 40 K. The blackbody mounting shelf and the main chamber have separate cold helium feeds with flow control valves to allow diversion of additional coolant to the blackbody, if needed. The cryoshields have several silicon diode thermometers attached at various points. These thermometers are monitored during calibration to ensure stability of the IR environment.

In order to provide a steady background flux for the ACR, a copper disk separates the blackbody chamber from the ACR chamber. This disk, called the isothermal plate, is moderately thermally insulated from the inner shield. The isothermal plate is fitted with an aperture that blocks the background radiation from the blackbody chamber without limiting the field of view of the ACR aperture. Complete isolation of the ACR chamber is achieved, if necessary, by a shutter in front of the isothermal plate that can be operated by remote control. The shutter is thermally anchored to the isothermal plate with a flexible copper braid. The temperature of the isothermal plate and shutter are monitored by three precision silicon diode thermometers. The isothermal plate is controlled to 0.05 K by using an integral heating element and the temperature is held at 22 K.

### 3.2 Measurements

**Fig. 2:**
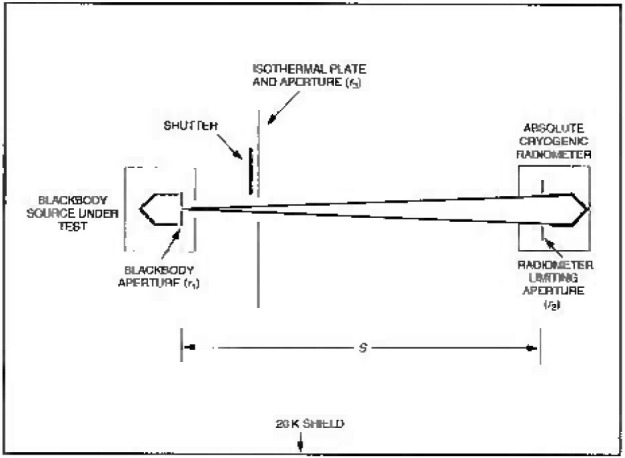
Blackbody calibration setup inside the LBIR chamber.

Figure 2 shows the calibration setup inside the LBIR chamber. Blackbody sensor data and radiant power data are collected at each blackbody temperature and aperture setting specified by the customer. Data are collected and recorded at approximately one second intervals for a period of three minutes at each setting. These data form the basis for estimating the repeatability of the instrumentation. Cryogenic preparation of the ACR and the complete calibration sequence are repeated on three different days to test the reproducibility of the entire measurement system.

#### 3.2.1 Blackbody Temperature Sensors

The blackbody temperature is generally measured by platinum resistance thermometers (PRTs) mounted on the blackbody core. Voltages across the PRTs measured by a multimeter, and currents supplied by a constant current source are converted to resistance values using Ohm’s law. The resistances are converted to temperature values if the customer provides data on the calibration of the PRT sensors. Nominal settings on the external temperature control electronics of the BB and measured temperatures for the BB sensors are shown in columns 1, 2, and 4 of Table 1, Experimental standard deviations from the 1 s repetitions at each setting on each day are shown in columns 3 and 5 of the same table.

#### 3.2.2 Radiant Power

The radiant power at the ACR aperture is measured as follows: The radiation from the blackbody is blocked from the ACR receiver by using the shutoff position on the blackbody aperture wheel (if available). The electrical heater power to the ACR receiver is set to a value higher (preferably 20% to 30%) than the expected radiant power from the blackbody so that the temperature controller servo (ac bridge) can maintain a constant temperature at the ACR cone by varying the amount of electrical power. Preliminary estimates of radiant power at the ACR aperture are made using the Stefan-Boltzmann law and the approximate distance between the ACR aperture and the blackbody aperture. After the ac bridge is balanced, the ACR is allowed to receive the blackbody radiation by turning the aperture wheel to a chosen aperture position. If the customer blackbody is equipped with a fixed aperture, the 152 mm (6 in) shutter in front of the isothermal plate is used for blocking the blackbody radiation. The difference between the initial electrical power setting with the shutter closed and the final electrical power setting with the shutter open is the radiant power at the ACR aperture. Data is taken continuously at one second intervals for at least 3 min with the shutter open, in order to determine the repeatability of the instrumentation.

#### 3.2.3 Geometry

The measurement of the radius of the blackbody aperture, *r*_1_ (see Fig. 2), corrected for thermal contraction at 20 K, is supplied by the customer. The radius of the ACR aperture, *r*_2_, has been measured by the Precision Engineering Division at NIST at room temperature in three different orientations. The average radius is corrected for thermal contraction as explained in Ref. [1].

The distance, S, between the blackbody aperture and the ACR limiting (precision) aperture is measured in two parts. The user measures the distance between the blackbody aperture and the front surface of a reference tab on the blackbody mounting plate as required by NIST [3], and NIST personnel measure the distance from the front surface of the tab to the ACR aperture. The chamber measurement is assisted by a Kaman proximity sensor [4] located internally to determine the final location of the reference tab to the ACR aperture before the chamber is evacuated. The measurements are made at ambient temperature and corrected for thermal contraction due to cooling of the chamber and the ACR to 20 K and 2 K, respectively. The radius of the aperture in the isothermal plate, *r*_3_ shown in Fig. 2, is chosen to limit the background flux from the blackbody front surface. However, it will allow the full cone of light from the blackbody aperture to reach the ACR aperture. The geometrical measurements for the BB are given in Table 2.

#### 3.2.4 Radiance Temperature

The following equation deduced from the Stefan-Boltzmann law is used to convert the radiant power to radiance temperature, *T*:

$$T={\left[\frac{P}{F_{1}A_{1}\sigma_{M}}\right]}^{1/4}$$ (1)

where

$$\begin{array}{l} F_{1}=(1/2)[z-{\left[z^{2}-4x^{2}\cdot y^{2}\right]}^{1/2}],\\ x=\frac{r_{2}}{S},y=\frac{S}{r_{1}},z=1+(1+x^{2})y^{2}, \end{array}$$

and *A*_1_ = π*r*_1_^2^ is the area of the blackbody aperture; *P* is the radiant power and *σ*_M_ is the Stefan-Boltzmann constant, and equal to 5.67051 × 10^−8^ W m^−2^ K^−4^. The expression for the configuration factor, F_1_, given above is taken from Ref. [5] and to a first order approximation reduces to π*r*_2_^2^/*s*^2^. The quantities *r*_1_, *r*_2_, and S have already been defined, and their measured values at room temperature are adjusted to cryogenic temperatures using the standard reference data for contraction of materials [6].

### 3.3 Analysis

#### 3.3.1 Diffraction Correction

It has been established in the literature [7, 8] that the radiation from the blackbody incident at the ACR aperture will not be solely determined according to geometrical optics because of diffraction effects at both limiting and nonlimiting apertures in the beam path. Therefore, diffraction losses at each one of the apertures in the beam path are estimated by using the procedures published in Refs. [7] and [8], The measured radiant power values and the deduced radiance temperatures are corrected for the total diffraction loss which is a product of the fractional losses at each aperture in the beam path, The calculated correction factors (Δ*P*/*P*) given as a percentage of the measured power values for the BB are listed in Table 3 for various temperatures. The corresponding correction factor (Δ*T*/*T*) for the radiance temperature is approximately 1/4 of the correction (Δ*P*/*P*) for the radiant power.

#### 3.3.2 Measurement Uncertainties

The uncertainties in the measurements are analyzed according to guidelines under development by the International Organization for Standardization (ISO) [9]. Uncertainties are caused by either systematic or random effects. Uncertainties from systematic effects (here after called systematic uncertainties for simplicity) are of the following types for this experiment: 1) uncertainties in theoretical corrections applied to measured data such as the uncertainties associated with the diffraction corrections; 2) uncertainties in the measured values of parameters that remain constant during the calibration. Examples of such uncertainties are the parameters that characterize the ACR response [1] which do not change during the ACR power measurements. These uncertainties are Type B uncertainties according to Ref. [9] Subclause 3.3.3. Uncertainties from random effects (hereafter called random uncertainties for simplicity) arise from the statistical variation in the measurements during the calibration experiment. This type of variation is inherent in the blackbody PRT sensor measurements of temperature and the ACR measurements of radiant power during the calibration experiment. These are Type A uncertainties [9].

##### Uncertainty in Radiant Power

The uncertainty in the ACR measurement of the radiant power is analyzed as follows: For each nominal temperature, radiant power data (*P*_1_) are collected every 1–1/2 s for approximately 3 min, leading to approximately 120 data points. An average, $\bar{P}$, and standard deviation, *S*_rpt_, are calculated from this data. The standard deviation, *S*_rpt_, is a measure of the repeatability of the measurements in each set, and in general, the ACR data show very good repeatability. The measurement process is repeated three different times to estimate a standard deviation, *S*_rpt_, for reproducibility. The average over the three runs is calculated as:

$$\bar{\bar{P}}=\frac{1}{3}\sum_{j=1}^{3}{\bar{P}}_{j}$$

a standard deviation (*S*_r_) Is calculated from

$$s_{r}^{2}=\sum_{j=1}^{3}{\left({\bar{P}}_{j}-\bar{\bar{P}}\right)}^{2}/2.$$ (4)

The standard deviation (*S*_r_) is a measure of both repeatability and reproducibility of measurements [10] according to

$$s_{t}^{2}\approx s_{rpr}^{2}+s_{rpt}^{2}/120.$$ (5)

Although more repetitions of the entire measurement process would be desirable for better statistics, time constraints limit the number of repetitions. The radiant powers, ${\bar{P}}_{j}$, and the average powers, $\bar{\bar{P}}$, for the BB are given in columns 6 and 7, respectively, of Table 1. The standard deviations of the means, $s_{t}/\sqrt{3}$, are given in column 8.

Two sources of systematic uncertainty are evaluated as Type B following Ref. [9]. The standard uncertainty (i.e., 1 *σ* level) in the diffraction correction to the measured radiant power is based on diffraction theory used to calculate the correction. This standard uncertainty is estimated to be ± 10% [7, 8] of the calculated correction based on scientific judgment (Sec. 4.6 Ref. [11]), as shown in column 3 of Table 3 for the BB. The standard uncertainty in the characterization of the radiometer as given in Ref. [1] is 0.12% of the measured radiant power. The standard uncertainty due to systematic effects is then the square root of the sum of the squares of the two components.

The random and systematic uncertainties are listed separately in the calibration report. The combined standard uncertainty (*u*_c_) is reported as the square root of the sum of the squares of all of the components. However, it should be noted that other methods of combining components of uncertainty from systematic and random effects are in practice for calculating the total uncertainty [12]. Therefore, all components of uncertainty are listed to give the customer the choice of using an alternative method.

The radiant power data from Table 1 after correction for diffraction loss are shown in column 6 of Table 4. The uncertainty components associated with the average power are shown in columns 7 and 8, and the combined standard uncertainty, *u*_c_, is given in column 9.

##### Uncertainly in the Biackbody Sensor Data

The Type A (random) uncertainty in the blackbody PRT sensor data is analyzed as follows: The PRT sensor data are collected in conjunction with the ACR power measurements. In general, the standard deviations for the PRT sensor data are homogeneous and negligibly small. Repeated sets of data are averaged to obtain the mean and the corresponding standard deviation analogously to the method discussed earlier for the ACR power measurements. The mean temperature values of the BB with associated standard deviations are shown in columns 2 through 5 of Table 4.

##### Radiance Temperature and Calibration Uncertainty

The final radiance temperatures for the calibration report are obtained by the least-squares analysis of temperatures, *T_ij_* (*i* = 1, …, *n*;*j* = 1, …,3), deduced from Eq. (1), as a function of blackbody sensor readings, *X_ij_* (*i* = 1, …, *n*; *j* = 1, …,3), for each aperture setting.

In order to evaluate a confidence band for the variability of the calibration curve, the following statistical procedure is adopted. The calibration equation is assumed to follow the model

$$T_{ij}=a_{0}+a_{⊥}X_{ij}+a_{2}X_{ij}^{2}+a_{3}X_{ij}^{3}+\ldots .+a_{k}X_{ij}^{k}+\epsilon_{ij},$$ (6)

where *a*_0_,…, *a_k_* are to be estimated, and *ϵ_ij_*, the random errors associated with the measurements *T_ij_*, are assumed to be independent with heterogeneous variances $\sigma_{ij}^{2}$. The *σ_ij_* are estimated by *s_ij_* from approximately 120 data points for each run. Weighted least squares [13] accounts for the heterogeneity of variances where the weights, *w_i_*, are calculated from the empirical variances by

$$w_{ij}=1/s_{ij}^{2}.$$ (7)

The standard deviations associated with the *T_ij_* are an order of magnitude larger than the standard deviations associated with the *X_ij_* data, and, therefore, random uncertainties associated with the measurements *X_ij_*, are assumed to be negligible for the purpose of the least squares analysis. The degree, *k*, for the polynomial in Eq. (6) is determined by a goodness-of-fit test [14] which compares the agreement among groups of three runs with the overall fit to the data. The lowest degree polynomial which satisfies the goodness-of-fit criterion is taken as the calibration curve.

Given a future blackbody sensor setting, *X_h_*, its calibrated radiance temperature value is given by

$$T_{h}={a^{\prime }}_{0}+{a^{\prime }}_{1}X_{h}+{a^{\prime }}_{2}X_{h}^{2}+{a^{\prime }}_{3}X_{h}^{3}\ldots .+{a^{\prime }}_{k}X_{h}^{k},$$ (8)

where ${a^{\prime }}_{0},\ldots ,{a^{\prime }}_{k}$ are least-squares estimates from calibration data. The random component of uncertainty associated with the predicted value is computed as

$$U_{\text{random}}={\left[\left(k+1)F(95;k+1,n-k-1\right)\right]}^{1/2}s(T_{h}).$$ (9)

The constant *F*(95;*k* + 1, *n* − *k* − 1) is the upper 95 percentile of Snedecor’s *F*-distribution with *k* +1 degrees of freedom in the numerator and *n* − *k* − 1 degrees of freedom in the denominator, and *s* (*T_h_*) is the standard deviation of the predicted value, *T_h_*. This uncertainty, based on the Working-Hoetelling confidence bands for the calibration curve, is valid for all future applications of the calibration curve as long as the model holds [15]. The details of the statistical analysis can be found in Ref. [15, 16]. A software package, OMNITAB [17], is used for the statistical computations.

Data for the least-squares analysis of the BB are shown in Table 5. Temperature as measured by PRT sensor 1 is used as the independent variable, *X*. The analysis confirms that a linear function (*k* = 1) is sufficient for describing the data, and the following equation

$$T_{h}={a^{\prime }}_{v}+{a^{\prime }}_{i}(X_{h})$$ (10)

gives the predicted radiance temperature (*T_h_*) for a blackbody temperature (*X_h_*) measured by PRT sensor 1. The estimated coefficients and associated standard deviations are:

$$\begin{array}{l} {a^{\prime }}_{0}=-1.096;s({a^{\prime }}_{0})=0.244\\ {a^{\prime }}_{1}=1.007;s({a^{\prime }}_{1})=0.001. \end{array}$$

The percentage uncertainty in the deduced radiance temperature, δ*T*/*T*, is given by Eq. (1) and the theory of uncertainty propagation [18]. An approximation on a Taylor series expression gives the relationship between the variables as

$$\delta T/T\sim \delta P/4P+\delta r_{t}/2r_{t}+\delta r_{2}/2r_{2}+\delta S/2S.$$ (11)

The first term on the right hand side of Eq. (11) shows the relationship to the contribution from the uncertainty in the radiant power which has both Type A and Type B components as discussed above. The last three terms in Eq. (11) show the relationship to the contributions from the uncertainties in the measurement of the geometrical quantities, *r*_1_, *r*_2_, and *S*. A summary of the percentage uncertainties in the geometrical measurements (Table 2) and their propagated uncertainties for radiance temperature measurements are given in Table 6.

The predicted NIST radiance temperature from the calibration equation for each average value of temperature measured by PRT sensor 1 is given in Table 7 for the BB calibration. The uncertainty components due to systematic effects are listed separately based on Eq. (11): 1) the uncertainty in the power *δP*/4*P*, due to the characterization of the ACR as an absolute detector [1] is listed in column 4; 2) the uncertainty in defining the geometry as given in Table 6 is shown in column 5; and 3) the uncertainty in diffraction calculations due to approximations is shown in column 6. The total systematic component, b, which is the root sum of squares, is given in column 7, The Type A component, *s*(*t_h_*), of the uncertainty is obtained from least-squares analysis, and the expanded uncertainty, *U*, as shown in column 9, is obtained by expanding Eq. (9) as follows [19]:

$$U={\left[\left(k+1)F(95;k+1,n-k-1\right)\right]}^{1/2}{\left[s^{2}(T_{h})+b^{2}\right]}^{1/2}.$$ (12)

The multiplying factor in Eq. (12) for *n* =27, *k* =1 for the BB experimental data is 2.6. The value shown in parenthesis in column 9 is the expanded uncertainly given as a percentage of the measured temperature.

The statistical procedure that leads to Eq. (12) did not take into account possible correlations between Type B uncertainties because of the relationship between variables shown in Eq. (11). However, calculations of expanded uncertainty, *U*, using the complete covariance matrix and least square fitting using appropriate weights have been carried out and the results are found to be the same as given by Eq. (12). The calibration constants in Eq. (10) also are found to be essentially same as reported earlier.

**Fig. 3:**
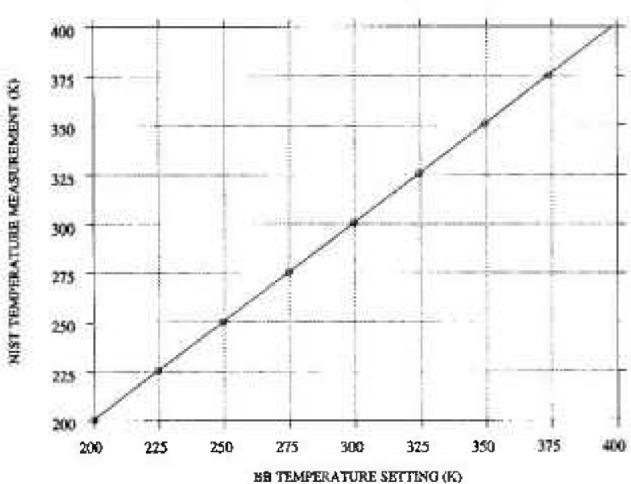
Measured temperatures and calibrated radiance temperature from polynomial fit plotted as a function of blackbody temperature setting using PRT sensor 1.

**Fig. 4:**
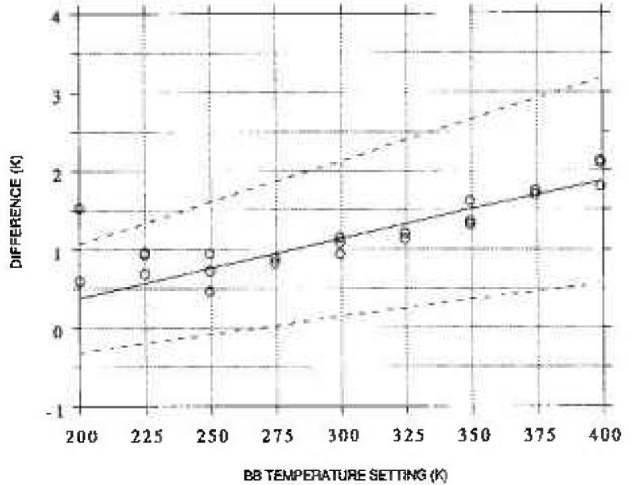
Solid line: Difference between the deduced radiance temperature and the temperature measured by the blackbody PRT sensor 1 plotted as a function of blackbody temperature setting. The points represent the same for measured radiance temperatures and are not all of equal weights. Dashed lines: Upper and lower bounds of 95% confidence bands.

Figure 3 shows the temperature value from PRT sensor 1 plotted on the *X*-axis and the measured radiance temperatures (Table 5) and corresponding predicted radiance temperature (Table 7) on the *Y*-axis. The solid line in Fig. 3 connects the predicted radiance temperature values and represents Eq. (10). Figure 4 shows the 95% confidence band for the difference between the calibrated temperature and the PRT temperature. The expanded uncertainty given in Table 7 is within 0.5%.

However, it is always an open question why for example, at the nominal blackbody temperature of the 400 K does the temperature sensor 1 measure 399.071 K whereas the radiance temperature is 400.9 K as shown in Table 7. The calibration curve does not address the effects of systematic errors such as unaccounted scattered light entering the ACR, aperture warming of the blackbody etc. However, experimental designs to eliminate such systematic effects are planned and implemented within the time allotted for customer calibrations. The experimental checks show these effects should be negligible. For future calibrations, spectral instrumentation is also being added to address this question further by measuring the emittance from the blackbody as a function of wavelength between 2 μm to 30 μm and comparing it with the currently assumed value of unity.

## 4. Summary

The LBIR facility is in operation for customer blackbody calibrations using the ACR as the absolute detector. The experimental procedure and analysis of data that generate the calibration report for the customer are described in detail, and a recent blackbody calibration at the facility serves as an example of the procedure. Calibration uncertainties in radiance temperatures of less than 1% have been realized for typical flux levels of 25 nW and above at the ACR aperture. Experimental procedures to reduce systematic uncertainties are continuously investigated and implemented to improve the accuracy of measurements. Future improvements of noise control in the ACR operation could allow measurements down to 10 nW at the ACR aperture with 1% uncertainty. Capability for measuring spectral emittance of customer sources in the 2 to 30 micrometer wavelength region is being developed.

## Figures and Tables

**Table 1:** Experimental data

Nominal (K)	Sensor 1 (K)	Std. dev. (K)	Sensor 2 CK)	Std. dev. (K)	Measured power (nW)	Average power (nW)	Std. dev. of average (nW)
200	199.897	0.002	199.281	0.002	71.9	71.5	0.5 (0.6%)
	199.874	0.005	199.332	0.005	72.0		
	199.947	0.002	199.328	0.003	70.6		
225	224.780	0.003	224.266	0.004	113.5	113.3	0.1 (0.1%)
	224.728	0.003	224.276	0.006	113.4		
	224.823	0.004	224.306	0.004	113.0		
250	249.662	0.004	249.268	0.004	171.3	172.0	0.4 (0.2%)
	249.639	0.007	249.288	0.008	172.6		
	249.733	0.005	249.322	0.004	172.2		
275	274.685	0.003	274.408	0.002	252.6	252.5	0.2 (0.1%)
	274.644	0.006	274.394	0.007	252.2		
	274.719	0.006	274.443	0.004	252.8		
300	299.531	0.007	299329	0.007	358.4	358.0	0.4 (0.1%)
	299.521	0.003	299.346	0.004	357.3		
	299.588	0.005	299.389	0.004	358.4		
325	324.412	0.005	324.346	0.006	497.6	494.6	1.4 (0.3%)
	324.457	0.005	324.375	0.005	492.8		
	324.446	0.003	324.379	0.004	493.4		
350	349.306	0.003	349.378	0.003	665.8	664.8	0.8 (0.1%)
	349.337	0.008	349.395	0.011	663.7		
	349.445	0.006	349.479	0.006	664.8		
375	374.047	0.005	374.284	0.005	882.2	878.3	2.0 (0.2%)
	374.069	0.003	374.292	0.003	875.7		
	374.171	0.006	374.377	0.007	877.0		
400	399.026	0.005	399.362	0.005	1138.0	1137.1	1.3 (0.1%)
	399.056	0.004	399.379	0.004	1134.6		
	399.131	0.004	399.444	0.003	1138.8		

**Table 2:** Geometric data for BB calibration

			Meas. value	Std. dev. mean
1. Radius (*r*_2_) of the ACR aperture at 2.2 K			1.4971 cm	5×10^−5^cm
2. Distance between the apertures (*S*)			30.77 cm	0.042 cm
3. BB aperture radius (*r*_1_):				
	
	Nominal mm	Measured at ambient mm	Deduced 20K mm	Std. dev. mean mm
	
	0.325	0.3251	0.3244	0.0013
	

**Table 3:** Calculated diffraction correction (Δ*P*) given as a percentage of the measured radiant power (*P*). Systematic uncertainty (Type B) in the correction due to approximations in the calculations is ±10% of Δ*P*/*P*.

Nominal blackbody temp. (K)	Diff. corr. Δ*P*/*P*	Uncer. (1 *σ*)
200	1.3%	±0.13%
225	1.2%	±0.12%
250	1.1%	±0.11%
275	1.0%	±0.1%
300	0.9%	±0.09%
325	0.8%	±0.08%
350	0.8%	±0.08%
375	0.7%	±0.07%
400	0.7%	±0.07%

**Table 4:** Average blackbody sensor data and radiant power corrected for diffraction effects at the ACR aperture for the BB

Nominal (K)	Sensor 1 data (K)	Std. dev. (κ)	Sensor 2 data (K)	Std. dev. (K)	Average power nW	Syst. uncer. nW	Random uncer. nW	Comh. uncer. *u*_c_(1*σ*) nW
200	199.906	0.003	199.313	0.003	72.4	0.1	0.5	0.5 (0.6%)
225	224.777	0.003	224.282	0.004	114.6	0.2	0.1	0.2 (0.2%)
250	249.678	0.005	249.293	0.005	173.9	0.3	0.4	0.5 (0.3%)
275	274.683	0.005	274.415	0.005	255.0	0.4	0.2	0.3 (0.2%)
300	299.547	0.005	299.355	0.005	361.2	0.5	0.4	0.6 (0.2%)
325	324.429	0.005	324.367	0.005	498.6	0.7	1.4	1.6 (0.3%)
350	349.362	0.006	349.429	0.007	669.8	1.0	0.8	1.3 (0.2%)
375	374.120	0.005	374.331	0.005	884.5	1.2	2.0	2.3 (0.3%)
400	399.071	0.005	399.395	0.004	1144.6	1.6	1.3	2.1 (0.2%)

**Table 5:** Data for the least-squares analysis of the BB

Nominal (K)	Sensor 1 (K) *X*	Radiance temperature (K) *T*	Standard deviation (K) *s*	Weight W
200	199.897	201.427	3.500	0.0798
	199.874^a^	201.539^a^	1.420	0.4957
	199.947	200.546	2.130	0.2203
225	224.780	225.736	2.990	0.1116
	224.728	225.666	1.384	0.5224
	224.823	225.506	1.975	0.2564
250	249.662	250.135	2.100	0.2278
	249.639	250.593	1.166	0.736
	249.733	250.463	1.865	0.2875
275	274.6849	275.575	1.102	0.8239
	274.644	275.466	1.319	0.5751
	274.719	275.605	1.101	0.8244
300	299.5309	300.683	0.715	1.9545
	299.5211	300.462	1.135	0.7760
	299.583	300.683	0.887	1.2702
325	324.412^a^	326.342^a^	0.830	1.4529
	324.457	325.600	0.603	2.7502
	324.446	325.658	1.076	0.8641
350	349.306	350.931	0.644	2.4084
	349.337	350.657	0.997	1.0056
	349.445	350.807	0.869	1.3233
375	374.047^a^	376.500^a^	0.660	2.2930
	374.069	375.766	0.816	1.5003
	374.171	375.915	1.407	0.4647
400	399.026	401.171	0.872	1.3164
	399.056	400.866	1.266	0.6242
	399.131	401.240	1.217	0.6748

a Data with standardized residuals from the linear fit exceeding 2.5. These outliers are not included in the final analysis.

**Table 6:** Uncertainties in the measurements of geometrical quantities for the BB

Quantity	Uncertainty in measurement (1*σ*)	Propagated uncer for temperature measurement (1 σ)
1) Distance between apertures (*δS*/*S*)	0.136%	0.068%
2) Radius of ACR aperture (*δr*_2_/*r*_2_)	0.003%	0.002%
3) Radius of BB aperture (*δr*_2_/*r*_2_)	0.2%·	0.1%
	Total^a^ 0.12%	

a Square root of sum of squares of 1, 2, and 3 given above.

**Table 7:** Predicted radiance temperatures and uncertainties for BB

Nominal	Sensor 1 data	Predicted radiance temp	ACR char.	Systematic uncertainties			1 σ *random* uncertainties	Expanded uncer. (*U*)
				Geometry meas.	Diff. cal.	Total *b*		
	(K)	(K)	(K)	(K)	(K)	(K)	(K)	(K)
200	199.906	200.3	0.06	0.24	0.07	0.25	0.10	0.7 (0.4%)
250	224.777	225.4	0.07	0.26	0.07	0.28	0.08	0.7 (03%)
250	249.678	250.4	0.08	0.30	0.08	0.32	0.07	0.7 (0.3%)
275	274.683	275.6	0.09	0.33	0.05	0.35	0.05	0.8 (0.3%)
300	299.547	300.7	0.09	0.36	0.06	0.38	0.04	0.8 (0.3%)
325	324.429	325.8	0.10	0.39	0.07	0.41	0.04	0.8 (0.3%)
350	349.362	350.9	0.11	0.42	0.07	0.44	0.04	0.9 (0.3%)
375	374.120	375.8	0.11	0.45	0.08	0.47	0.05	1.0 (0.3%)
400	399.071	400.9	0.12	0.48	0.08	0.5	0.07	1.0 (0.3%)

## Footnotes

1 Certain commercial equipment, instruments, or materials are identified in this paper to specify adequately the experimental procedure. Such identification does not imply recommendation or endorsement by the National Institute of Standards and Technology, nor does it imply that the materials or equipment identified are necessarily the best available for the purpose.
